# Supplementary material for: MetaRibo-Seq measures translation in microbiomes
Source: Nat Commun. 2020 Jun 29;11:3268. doi: 10.1038/s41467-020-17081-z (PMC7324362; doi:10.1038/s41467-020-17081-z)
Supplement: Supplementary file 10 — Supplementary Data 7 [file 41467_2020_17081_MOESM10_ESM.zip › File2/Confidence_High_Taxonomy/177813_out.krona.html]

Javascript must be enabled to view this page.

members
magnitude
magnitudeUnassigned
count
unassigned
taxon
rank

177813\_out

206

superkingdom
206
2

1239
phylum
206

206
class
186801

186802
206
order

190
family
31979

genus

SRS019968\_contig\_number\_12241SRS020233\_contig\_number\_contig-100\_6845.229636SRS054956\_contig\_number\_contig-100\_35912.35912SRS058770\_contig\_number\_contig-100\_28229.68246SRS147919\_contig\_number\_contig-100\_25161.25161SRS148159\_contig\_number\_27514SRS148319\_contig\_number\_contig-100\_8840.50990
580596
189
7


SRS013687\_contig\_number\_31570SRS014235\_contig\_number\_46386SRS014459\_contig\_number\_31708SRS014923\_contig\_number\_contig-100\_2046.220185SRS015264\_contig\_number\_contig-100\_2438.2438SRS015960\_contig\_number\_contig-100\_805.132689SRS017307\_contig\_number\_27936SRS017916\_contig\_number\_14836SRS018888\_contig\_number\_contig-100\_1209.1210SRS019381\_contig\_number\_contig-100\_1113.128391SRS019397\_contig\_number\_12754SRS019808\_contig\_number\_20851SRS022071\_contig\_number\_35557SRS024663\_contig\_number\_15696SRS046502\_contig\_number\_14080SRS049402\_contig\_number\_2585SRS049959\_contig\_number\_contig-100\_362.293819SRS050998\_contig\_number\_5500SRS053214\_contig\_number\_2349SRS063190\_contig\_number\_contig-100\_86.72686SRS063813\_contig\_number\_1705SRS064276\_contig\_number\_39292SRS075773\_contig\_number\_39026SRS076976\_contig\_number\_13179SRS078419\_contig\_number\_35463SRS097889\_contig\_number\_29737SRS098061\_contig\_number\_9890SRS1041091\_contig\_number\_11857SRS1041138\_contig\_number\_14835SRS1041141\_contig\_number\_10492SRS104693\_contig\_number\_4398SRS104912\_contig\_number\_21577SRS104975\_contig\_number\_17478SRS105082\_contig\_number\_17182SRS105153\_contig\_number\_38924SRS142599\_contig\_number\_2224SRS143372\_contig\_number\_9328SRS143780\_contig\_number\_3396SRS144506\_contig\_number\_49612SRS144537\_contig\_number\_31643SRS147022\_contig\_number\_14029SRS148784\_contig\_number\_19457SRS149075\_contig\_number\_18234SRS149181\_contig\_number\_25413SRS150029\_contig\_number\_contig-100\_1501.103631SRS893270\_contig\_number\_23978SRS893358\_contig\_number\_14737SRS893373\_contig\_number\_25511
species
48
1828555


SRS011134\_contig\_number\_30596SRS011405\_contig\_number\_contig-100\_7087.93434SRS012849\_contig\_number\_contig-100\_434.200631SRS013098\_contig\_number\_23404SRS013521\_contig\_number\_1535SRS014415\_contig\_number\_2018SRS014613\_contig\_number\_contig-100\_1227.124927SRS014736\_contig\_number\_2614SRS015065\_contig\_number\_36048SRS015190\_contig\_number\_contig-100\_8530.76747SRS015431\_contig\_number\_27955SRS015579\_contig\_number\_14765SRS015782\_contig\_number\_43654SRS016840\_contig\_number\_contig-100\_2434.2435SRS017375\_contig\_number\_contig-100\_150.151SRS017521\_contig\_number\_40329SRS018351\_contig\_number\_3800SRS018817\_contig\_number\_23728SRS018836\_contig\_number\_contig-100\_1258.164339SRS019267\_contig\_number\_contig-100\_4329.35372SRS019285\_contig\_number\_495SRS019445\_contig\_number\_36639SRS019496\_contig\_number\_contig-100\_13.109103SRS020394\_contig\_number\_contig-100\_9.110107SRS021219\_contig\_number\_13547SRS024625\_contig\_number\_1181SRS043768\_contig\_number\_contig-100\_825.134748SRS043841\_contig\_number\_4141SRS044535\_contig\_number\_1998SRS045004\_contig\_number\_30729SRS045645\_contig\_number\_contig-100\_1166.169712SRS045713\_contig\_number\_12749SRS045826\_contig\_number\_7500SRS046369\_contig\_number\_8637SRS047014\_contig\_number\_22450SRS047044\_contig\_number\_8299SRS047433\_contig\_number\_23791SRS049446\_contig\_number\_10863SRS049712\_contig\_number\_24012SRS049773\_contig\_number\_35976SRS050752\_contig\_number\_contig-100\_2931.161741SRS051031\_contig\_number\_contig-100\_7535.142688SRS051610\_contig\_number\_10587SRS053356\_contig\_number\_30723SRS053573\_contig\_number\_contig-100\_3123.31183SRS054059\_contig\_number\_5121SRS056259\_contig\_number\_25420SRS056273\_contig\_number\_34360SRS058070\_contig\_number\_contig-100\_906.181429SRS075078\_contig\_number\_28964SRS075341\_contig\_number\_24268SRS077194\_contig\_number\_24007SRS077231\_contig\_number\_contig-100\_585.105763SRS077641\_contig\_number\_20047SRS098073\_contig\_number\_contig-100\_1297.1298SRS098571\_contig\_number\_39200SRS101376\_contig\_number\_contig-100\_162.163673SRS1041036\_contig\_number\_5424SRS1041039\_contig\_number\_3282SRS1041137\_contig\_number\_31895SRS1041139\_contig\_number\_6713SRS104311\_contig\_number\_42925SRS104485\_contig\_number\_4848SRS143085\_contig\_number\_contig-100\_9.49062SRS143598\_contig\_number\_22938SRS143876\_contig\_number\_3021SRS144183\_contig\_number\_20671SRS146764\_contig\_number\_26849SRS146813\_contig\_number\_5585SRS147346\_contig\_number\_64228SRS147557\_contig\_number\_22975SRS148196\_contig\_number\_16272SRS148874\_contig\_number\_16988SRS892882\_contig\_number\_8777SRS893170\_contig\_number\_9986SRS893295\_contig\_number\_11173SRS893369\_contig\_number\_contig-100\_7.76590SRS971275\_contig\_number\_18579
78
species
2292294

2292293
56
species

SRS011084\_contig\_number\_13817SRS013476\_contig\_number\_30050SRS014412\_contig\_number\_18913SRS014683\_contig\_number\_17518SRS015590\_contig\_number\_13081SRS015890\_contig\_number\_26208SRS017701\_contig\_number\_contig-100\_581.90510SRS018541\_contig\_number\_10454SRS018656\_contig\_number\_12250SRS018984\_contig\_number\_32622SRS019030\_contig\_number\_8258SRS019068\_contig\_number\_113492SRS019685\_contig\_number\_contig-100\_1177.217185SRS019693\_contig\_number\_5968SRS020271\_contig\_number\_3921SRS021484\_contig\_number\_30510SRS022137\_contig\_number\_contig-100\_681.124676SRS022609\_contig\_number\_51976SRS024009\_contig\_number\_12238SRS024132\_contig\_number\_contig-100\_1911.269136SRS024435\_contig\_number\_39786SRS043411\_contig\_number\_17159SRS046717\_contig\_number\_4926SRS049896\_contig\_number\_31951SRS050941\_contig\_number\_contig-100\_10760.98166SRS053649\_contig\_number\_22162SRS055017\_contig\_number\_3473SRS055982\_contig\_number\_19682SRS063518\_contig\_number\_34563SRS065397\_contig\_number\_15210SRS075984\_contig\_number\_15521SRS076804\_contig\_number\_20891SRS077127\_contig\_number\_17928SRS077392\_contig\_number\_34667SRS101433\_contig\_number\_15569SRS103987\_contig\_number\_33911SRS104036\_contig\_number\_6497SRS104084\_contig\_number\_14729SRS1041032\_contig\_number\_13770SRS1041095\_contig\_number\_contig-100\_146.34243SRS1055043\_contig\_number\_14921SRS1055067\_contig\_number\_10302SRS1055083\_contig\_number\_contig-100\_904.82442SRS1055099\_contig\_number\_1541SRS143181\_contig\_number\_2469SRS145308\_contig\_number\_4288SRS145497\_contig\_number\_16597SRS147271\_contig\_number\_31891SRS147614\_contig\_number\_883SRS148424\_contig\_number\_35884SRS893259\_contig\_number\_406SRS893292\_contig\_number\_7741SRS893300\_contig\_number\_contig-100\_1358.76696SRS893366\_contig\_number\_19170SRS893378\_contig\_number\_22491SRS971276\_contig\_number\_31790

genus
1
1485

1650661

SRS147139\_contig\_number\_contig-100\_28743.61313
species
1

186803
family
2

1
genus
841

1262947
1
species

SRS140492\_contig\_number\_28450

1
species

SRS050520\_contig\_number\_contig-100\_404.405
658087

2
family
216572

459786
genus
2

species
1

SRS098881\_contig\_number\_2554
1897011


SRS075878\_contig\_number\_11438
1
species
876091

family
12
541000

species
12

SRS015663\_contig\_number\_contig-100\_455.201020SRS016095\_contig\_number\_21701SRS020869\_contig\_number\_1773SRS042628\_contig\_number\_8987SRS053398\_contig\_number\_23866SRS054352\_contig\_number\_24125SRS1041116\_contig\_number\_9053SRS142542\_contig\_number\_24929SRS143895\_contig\_number\_contig-100\_490.263897SRS144362\_contig\_number\_24211SRS144603\_contig\_number\_14508SRS148817\_contig\_number\_contig-100\_2182.74880
552398
